# Assessing the Impact of Simulation-Based Learning on Student Satisfaction and Self-Confidence in Critical Care Medicine

**DOI:** 10.1155/2024/6908005

**Published:** 2024-10-18

**Authors:** Mohammed Ageel

**Affiliations:** Department of Surgery, College of Medicine, Jazan University, Jazan, Saudi Arabia

**Keywords:** critical care medicine, medical education, self-confidence, simulation-based training, student satisfaction

## Abstract

**Background:** Simulation-based learning (SBL) is increasingly used in medical education to prepare students for clinical practice. This study aimed to evaluate the satisfaction and self-confidence of final-year medical students after attending SBL in critical care medicine.

**Methods:** A cross-sectional study was conducted among 117 final-year medical students at Jazan University, Saudi Arabia. Participants attended SBL sessions focusing on critical care skills and scenarios. Data were collected using a self-administered questionnaire, which included demographic information and the Student Satisfaction and Self-Confidence in Learning Scale. Statistical analyses included descriptive and inferential statistics.

**Results:** The study population comprised 61.54% females and 38.46% males, with a majority aged 24 years. Students who rated their overall learning experience as “Good” showed significantly higher satisfaction (4.20 ± 0.77) and self-confidence (4.20 ± 0.69) scores. The overall mean scores for satisfaction and self-confidence were 3.71 ± 0.88 and 3.70 ± 0.87, respectively. A strong positive correlation (*p* < 0.001) was found between satisfaction and self-confidence levels. The highest satisfaction and self-confidence scores were associated with the variety of learning materials and the instructors' helpfulness.

**Conclusion:** The SBL intervention was effective in enhancing student satisfaction and self-confidence in critical care medicine. The strong correlation between satisfaction and self-confidence highlights the importance of well-designed SBL programs in preparing medical students for clinical practice in critical care settings.

## 1. Introduction

The field of critical care medicine is characterized by high-stakes situations that demand rapid decision-making, effective communication, and proficient clinical skills from healthcare providers [1–3]. Simulation-based learning (SBL) has emerged as a valuable educational approach in preparing medical students for the challenges of critical care practice [4, 5].

SBL offers a controlled and realistic environment where students can safely acquire and refine essential competencies without compromising patient safety [6]. Through simulated scenarios, students can experience rare and critical events, practice complex procedures, and develop teamwork and communication abilities crucial in managing critically ill patients [3].

One of the primary objectives of SBL in critical care medicine is to enhance students' self-confidence and satisfaction with their learning experience. Self-confidence is a vital attribute for healthcare professionals, enabling them to make informed decisions, communicate effectively, and perform clinical tasks with assurance [6]. A lack of self-confidence can lead to hesitation, indecision, and potentially compromised patient care. Student satisfaction, on the other hand, is a key indicator of the effectiveness and acceptability of educational interventions [7]. Satisfaction with the learning experience is defined as the learners' overall contentment and positive perception of the SBL experience in anesthesiology, encompassing aspects such as the effectiveness of teaching methods, suitability of learning materials, and enjoyment derived from the instructor's teaching style. When students are satisfied with their learning experience, they are more likely to engage actively, retain information, and develop a positive attitude towards the subject matter. Conversely, dissatisfaction can lead to disengagement, poor performance, and a negative perception of the learning environment [8–10].

In the context of medical education in Saudi Arabia, SBL has been increasingly adopted as a valuable pedagogical tool in critical care medicine. However, there is a need for comprehensive research to evaluate the impact of SBL on students' satisfaction and self-confidence, particularly among final-year medical students who are on the cusp of transitioning to clinical practice [11]. The aim of this study is to evaluate students' satisfaction and self-confidence after attending SBL in critical care medicine using skills labs and hands-on sessions.

## 2. Materials and Methods

### 2.1. Study Design and Population

This cross-sectional study was conducted from January to December 2023 to evaluate the satisfaction and confidence of final-year medical students. The study population consisted of all 117 final-year medical students enrolled at Jazan University's College of Medicine, Saudi Arabia. The questionnaire was administered immediately after the students completed the SBL sessions to capture their perceptions and experiences.

### 2.2. Ethical Considerations

The study adhered to the principles outlined in the Declaration of Helsinki, and ethical approval was obtained from the Institutional Review Board of Jazan University. Participants were assured of anonymity and confidentiality throughout the research process, and written informed consent was obtained before their participation.

### 2.3. SBL Intervention

The SBL intervention was designed to enhance critical care medicine competencies, focusing on the management of critically ill patients, rapid response to emergencies, and effective communication within multidisciplinary teams. The curriculum was developed in collaboration with experienced critical care physicians and educational specialists, incorporating high-fidelity simulation technology and realistic clinical scenarios. The SBL was administered over 2 months, with participants engaging in two-hour weekly sessions.

Skills Lab Sessions aimed to develop procedural skills essential in critical care settings, such as advanced airway management, vascular access, and cardiopulmonary resuscitation (CPR). Students practiced these techniques using low to medium-fidelity simulators under the guidance of faculty members. The following procedures were covered: Basic Life Support (BLS) Simulation involved training participants in the fundamental aspects of BLS, including the recognition of cardiac arrest, activation of emergency response systems, and initiation of high-quality chest compressions. Proper hand placement, compression depth, and rate were emphasized to ensure effective CPR. Airway Management included techniques such as the head-tilt-chin-lift and jaw-thrust maneuvers to ensure a clear airway. Participants learned to provide effective ventilation using a bag valve mask, focusing on proper seal and ventilation rate. The correct sizing and insertion of oropharyngeal airways were demonstrated and practiced. Trainees were instructed on the use of a laryngoscope to visualize the vocal cords and facilitate endotracheal intubation. The procedure for inserting a Laryngeal Mask Airway (LMA) was covered as an alternative to endotracheal intubation. Various methods of oxygen delivery, including nasal cannulas, simple face masks, and non-rebreather masks, were discussed and practiced. An introduction to mechanical ventilation settings, modes, and troubleshooting was provided. The use of a crash cart, including defibrillation techniques and medication administration, was demonstrated. Techniques for establishing peripheral IV access were practiced using simulation arms. The procedure for inserting Central Venous Catheters (CVCs) was taught using a central venous catheterization simulator.

A comprehensive array of simulation tools and equipment was utilized to enhance the learning experience. These included a Portable Colour Doppler Ultrasound Machine, Blood Pressure Simulator and Arm, Cardiology Patient Simulator “K Plus,” E-Scope II Electronic Stethoscope and Traditional Stethoscope, Arterial Puncture Arm and Advanced Injection Training Arms, Negroid Injection Training Arm and I.V. Injection Simulator, Nasogastric Tube Feeding Model, Intubation Dummy and Laryngoscope Set, METI Emergency Care Simulator, Central Venous Catheterization Simulator, Cricothyrotomy Simulator (Neck and Thyroid Gland Exam Trainer), Sonorio with FAST Ultrasound for the TREUMAwan System, Femora Line Man Training Package, Paracentesis Ultrasound Training Model, Thoracic Trauma Trainer, Pericardiocentesis Simulator, Child and Adult Airway Management Kits, Thoracocentesis Model (Pleural Drainage Manikin), Infant Airway and Baby Anne TM 4-Pack, Neo-Natal Model for Intubation Practice, and PEDI Blue Neonatal Simulator with Smartskin Technology.

High-fidelity simulation scenarios were employed to mimic real-life critical care situations, such as managing septic shock, acute respiratory distress syndrome, or cardiac emergencies. These exercises emphasized teamwork, decision-making, and the application of clinical knowledge in a simulated intensive care unit environment. Participants were required to respond to dynamic and evolving scenarios, enhancing their ability to think critically and act swiftly in real clinical settings. The integration of these comprehensive SBL interventions aimed to equip participants with the necessary skills and confidence to manage critical care situations effectively, ultimately improving patient outcomes and fostering a culture of excellence in clinical practice.

### 2.4. Data Collection Tool

A self-administered structured questionnaire was used for data collection, comprising socio-demographic factors and the previously validated Students' Satisfaction and Self-confidence Scale [12, 13]. This scale has been used in multiple studies to assess student satisfaction and self-confidence in SBL environments [14, 15]. The socio-demographic factors included age, gender, and an overall rating of the learning experience (ranging from poor to good). The Student Satisfaction and Self-Confidence in Learning instrument consists of 13 items, designed to measure student satisfaction (five items) with the simulation activity and self-confidence in learning (eight items), using a five-point Likert-type scale ranging from strongly disagree to strongly agree. The questionnaire was administered in English and was made available to students through a unique bar-code linked to a Google Form. Students were instructed to scan the bar-code using their mobile phones to access the questionnaire.

In the current study, the reliability coefficient for student satisfaction and self-confidence in learning scale was conducted. The Cronbach's alpha coefficients were calculated to assess the internal consistency of the scale. The results indicated a Cronbach's alpha of 0.89 for the satisfaction component and 0.87 for the self-confidence component. These values suggest that the scale is highly reliable for measuring the intended constructs in the context of our study.

### 2.5. Data Analysis

The Statistical Package for Social Sciences (SPSS) version 26 software (IBM Corp., Armonk, N.Y., USA) was used for data analysis. Descriptive statistics were employed to evaluate the demographic data and means, while frequency distributions were used to summarize the responses to the questionnaire. Moreover, non-parametric tests were used to analyze the data as the variables did not meet the assumptions of normality. The Mann–Whitney U Test and the Kruskal–Wallis one-way ANOVA were conducted to examine the association between different variables. A Spearman correlation analysis was performed to investigate the relationship between student satisfaction and self-confidence. Statistical significance was set at a *p* value of 0.05.

## 3. Results

The study's demographic analysis included 117 final-year medical students, with a distribution of 61.54% females (*n* = 72) and 38.46% males (*n* = 45). Age-wise, 35.04% were 23 years old (*n* = 41), 40.17% were 24 years old (*n* = 47), and 24.79% were 25 years or older (*n* = 29). Upon assessing their learning experience, 44.44% (*n* = 52) rated it as “Good,” 47.86% (*n* = 56) as “Average,” and 7.69% (*n* = 9) as “Poor.” A significant difference was noted in satisfaction and self-confidence scores in relation to the learning experience rating, with “Good” ratings corresponding to higher mean satisfaction (4.20 ± 0.77) and self-confidence (4.20 ± 0.69) scores (*p* < 0.001). No significant differences were observed in satisfaction and self-confidence based on gender or age (*p* > 0.05) (Table 1).

The evaluation of student satisfaction with the SBL intervention showed that the majority of participants agreed that the teaching methods and materials were effective. The mean satisfaction scores for the items ranged from 3.68 ± 0.96 to 3.75 ± 0.9. The highest mean satisfaction score was for the item “The skills lab and hands-on sessions provided me with a variety of learning materials and activities to promote my learning” (3.75 ± 0.9, 60.69% agreement), followed by “My instructors used helpful resources to teach the skills lab and hands-on sessions” (3.75 ± 0.98, 65.82% agreement) (Table 2).

In terms of self-confidence, participants reported high confidence levels in mastering the content and skills presented during the SBL sessions. The mean self-confidence scores for the items related to content mastery, critical content coverage, and skill development in a clinical setting ranged from 3.59 ± 1.01 to 3.85 ± 1.13. The item with the highest mean self-confidence score was “It is the instructor's responsibility to tell me what I need to learn from the skills lab and hands-on session activity content during class time” (3.85 ± 1.13, 65.81% agreement), and the lowest was “I am confident that I am mastering the content of the skills lab and hands-on sessions activity that my instructors presented to me” (3.59 ± 1.01, 56.41% agreement) (Table 2).

**Figure 1:**
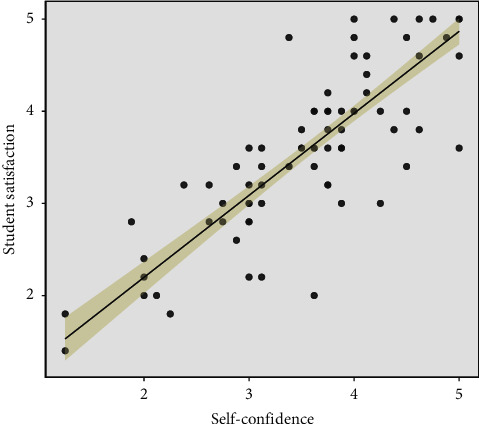
Correlation between student satisfaction and self-confidence in simulation-based learning.

The overall mean score for student satisfaction with the SBL intervention was 3.71 ± 0.88. The distribution of satisfaction scores was as follows: 7.69% (*n* = 9) in the lower interval, 41.03% (*n* = 48) in the intermediate interval, and 51.28% (*n* = 60) in the higher interval. Similarly, the overall mean score for self-confidence was 3.70 ± 0.87, with 6.84% (*n* = 8) in the lower interval, 37.61% (*n* = 44) in the intermediate interval, and 55.56% (*n* = 65) in the higher interval (Table 3). A significant positive correlation was found between the levels of satisfaction and self-confidence (*R* = 0.88, *p* < 0.001) (Figure 1).

## 4. Discussion

Obtaining student feedback is a cornerstone of effective medical education, offering insights into learner experiences and shaping future curricular improvements [16, 17]. In high-stakes domains like critical care, assessing satisfaction and self-confidence is particularly important. These metrics reveal student perceptions of their preparedness to manage complex and potentially life-threatening situations [18, 19]. This study explored the impact of an SBL intervention on medical students' satisfaction and self-confidence in the context of critical care medicine.

The findings of this study underscore the significant impact of SBL on enhancing student satisfaction and self-confidence among final-year medical students in critical care settings. Our results demonstrated a generally positive learning experience, with favorable ratings for both satisfaction and self-confidence. Participants reported overall satisfaction with the intervention (overall mean satisfaction score: 3.71 ± 0.88) and expressed greater self-confidence in their abilities (overall mean self-confidence score: 3.70 ± 0.87). The overall high satisfaction and self-confidence scores reported in this study align with previous research, which has demonstrated that SBL can effectively improve both cognitive and psychomotor skills in medical education [20, 21]. The positive correlation between satisfaction and self-confidence (*p* < 0.001) aligns with prior research [21] and illuminates a key principle: students who feel positively engaged in the educational process are more likely to develop confidence in their abilities. This enhanced self-confidence could translate into improved decision-making and skill execution under the pressures of critical care environments.

Critical care medicine requires a high level of proficiency in technical skills and decision-making under pressure. The high scores in self-confidence observed in this study, particularly in items related to mastering content and developing necessary skills, indicate that SBL is an effective method for preparing students for the complexities of critical care environments. This supports the findings of a previous study, which found that simulation training significantly improved the decision-making and critical thinking abilities of medical students in emergency and critical care scenarios [22].

Moreover, the significant association between the ratings of the learning experience and the levels of satisfaction and self-confidence highlights the importance of the quality of educational delivery in SBL. Students who rated their learning experience as “Good” demonstrated markedly higher satisfaction and confidence levels. This emphasizes the need for well-structured and high-quality simulation programs to optimize learning outcomes, a point echoed by a previous study, which emphasized that the educational design of simulation activities must be carefully planned and executed to achieve desired educational outcomes [6].

The study also revealed that the variety of learning materials and the helpfulness of resources provided by instructors were highly valued by the students, which contributed to their overall satisfaction and confidence. This finding is in line with the principles of adult learning theory, which posits that adult learners benefit from a variety of learning methods and practical applications of theoretical knowledge [23]. It also supports the notion that effective learning in medical education, particularly in high-stakes fields like critical care, requires diverse educational tools and resources to cater to different learning styles and needs [24].

However, the lower confidence levels associated with mastering the content presented by instructors, despite being positive; suggest that there may be areas within the SBL curriculum that require further enhancement. This aspect of the findings indicates the necessity for ongoing curriculum assessment and revision to ensure that all educational content is effectively communicated and understood by students. Continuous improvement of educational strategies is crucial to maintain the relevance and effectiveness of simulation-based education in adapting to the evolving needs of medical training [25].

### 4.1. Limitations

This study has several limitations that should be considered when interpreting the results. Although the study utilized a previously validated Students' Satisfaction and Self-confidence Scale, the assessment of student satisfaction was based on only five items, which may not fully capture the complexity of this concept in simulation-based anesthesiology training. The study was conducted with a single class of final-year medical students at one university, limiting the generalizability of the findings. The cross-sectional design provides only a snapshot of student satisfaction and self-confidence at a single point in time.

## 5. Conclusion

This study provides compelling evidence that SBL in critical care medicine significantly enhances student satisfaction and self-confidence. These findings advocate for the continued use and development of SBL programs in medical education, particularly in specialties that demand high levels of clinical competence and decision-making capabilities.

## Figures and Tables

**Table 1:** Demographic profile, learning experience ratings, and mean scores of satisfaction and self-confidence among of final-year medical students.

Variables	Variables	Count (%)	Satisfaction mean ± SD	*p* value	Self conf. mean ± SD	*p* value
Gender	Female	72 (61.54%)	3.79 ± 0.81	0.248	3.69 ± 0.84	0.915
	Male	45 (38.46%)	3.56 ± 0.98		3.70 ± 0.92	

Age	23	41 (35.04%)	3.62 ± 0.99	0.768	3.66 ± 1.00	0.923
	24	47 (40.17%)	3.72 ± 0.80		3.68 ± 0.82	
	≥25	29 (24.79%)	3.81 ± 0.85		3.77 ± 0.76	

Rate your learning experience	Good	52 (44.44%)	4.20 ± 0.77	<0.001	4.20 ± 0.69	<0.001
	Average	56 (47.86%)	3.34 ± 0.74		3.35 ± 0.77	
	Poor	9 (7.69%)	3.16 ± 0.91		2.89 ± 0.75	

**Table 2:** Participants' responses on student satisfaction and self-confidence in learning items.

Items	Disagreement	Neutral	Agreement	Mean ± SD
Student satisfaction				
1. The teaching methods used in these courses' (skills lab and hand on sessions) were helpful and effective	13 (11.11%)	33 (28.21%)	71 (60.69%)	3.69 ± 0.96
2. The skills lab and hand on sessions provided me with a variety of learning materials and activities to promote my learning	10 (8.55%)	36 (30.77%)	71 (60.69%)	3.75 ± 0.9
3. I Enjoyed how my instructor taught the skills lab and hand on sessions	14 (11.96%)	33 (28.21%)	70 (59.83%)	3.68 ± 0.96
4. The teaching materials used in these skills lab and hand on sessions were motivated and helped me to learn	14 (11.96%)	33 (28.21%)	70 (59.83%)	3.71 ± 1.08
5. The way my instructor taught the skills lab and hand on sessions was suitable to the way I learn	13 (11.11%)	33 (28.21%)	71 (60.68%)	3.69 ± 0.99
Self-confidence in learning				
6. I am confident that I am mastering the content of the skills lab and hand on sessions activity that my instructors presented to me	17 (14.53%)	34 (29.06%)	66 (56.41%)	3.59 ± 1.01
7. I am confident that this skills lab and hand on sessions covered critical content necessary for mastery of the curriculum	15 (12.82%)	29 (24.79%)	73 (62.40%)	3.68 ± 1.07
8. I am confident that I am developing the skills and obtaining the required knowledge from this skills lab and hand on sessions to perform necessary tasks in a clinical setting	17 (14.53%)	27 (23.08%)	73 (62.39%)	3.68 ± 1.01
9. My instructors used helpful resources to teach the skills lab and hand on sessions	14 (11.97%)	26 (22.22%)	77 (65.82%)	3.75 ± 0.98
10. It is my responsibility as a student to learn what I need to know from this skills lab and hand on sessions activity	17 (14.53%)	34 (29.06%)	66 (56.41%)	3.62 ± 1.11
11. I know how to get help when I do not understand the concept covered in the skills lab and hand on sessions	12 (10.25%)	29 (24.79%)	76 (64.96%)	3.79 ± 1.01
12. I know how to use the skills lab and hand on session activities to learn critical aspects of these skills	17 (14.53%)	33 (28.21%)	67 (57.26%)	3.59 ± 1.06
13. It is the instructor's responsibility to tell me what I need to learn from the skills lab and hand on session activity content during class time	15 (12.82%)	25 (21.37%)	77 (65.81%)	3.85 ± 1.13

**Table 3:** Overall mean scores and distribution of student satisfaction and self-confidence in learning.

	Mean ± SD	Lower interval	Intermediate interval	Higher interval
Student satisfaction	3.71 ± 0.88	9 (7.69%)	48 (41.03%)	60 (51.28%)
Self-confidence	3.70 ± 0.87	8 (6.84%)	44 (37.61%)	65 (55.56%)

## Data Availability

The data that support the findings of this study are available from the corresponding author upon reasonable request.
